# Partial Atomic Tin Nanocomplex Pillared Few-Layered Ti_3_C_2_T_x_ MXenes for Superior Lithium-Ion Storage

**DOI:** 10.1007/s40820-020-0405-7

**Published:** 2020-03-25

**Authors:** Shunlong Zhang, Hangjun Ying, Bin Yuan, Renzong Hu, Wei-Qiang Han

**Affiliations:** 1 School of Materials Science and Engineering, Zhejiang University, 310027 Hangzhou, People’s Republic of China; 2 School of Materials Science and Engineering, South China University of Technology, 510641 Guangzhou, People’s Republic of China; 3 Key Laboratory of Advanced Energy Storage Materials of Guangdong Province, 510641 Guangzhou, People’s Republic of China

**Keywords:** Pillared MXene, Few-layered MXene, Tin nanocomplex, Lithium-ion storage

## Abstract

MXenes have attracted great interest in various fields, and pillared MXenes open a new path with larger interlayer spacing. However, the further study of pillared MXenes is blocked at multilayered state due to serious restacking phenomenon of few-layered MXene nanosheets. In this work, for the first time, we designed a facile NH^4+^ method to fundamentally solve the restacking issues of MXene nanosheets and succeeded in achieving pillared few-layered MXene. Sn nanocomplex pillared few-layered Ti_3_C_2_T_x_ (STCT) composites were synthesized by introducing atomic Sn nanocomplex into interlayer of pillared few-layered Ti_3_C_2_T_x_ MXenes via pillaring technique. The MXene matrix can inhibit Sn nanocomplex particles agglomeration and serve as conductive network. Meanwhile, the Sn nanocomplex particles can further open the interlayer spacing of Ti_3_C_2_T_x_ during lithiation/delithiation processes and therefore generate extra capacity. Benefiting from the “pillar effect,” the STCT composites can maintain 1016 mAh g^−1^ after 1200 cycles at 2000 mA g^−1^ and deliver a stable capacity of 680 mAh g^−1^ at 5 A g^−1^, showing one of the best performances among MXene-based composites. This work will provide a new way for the development of pillared MXenes and their energy storage due to significant breakthrough from multilayered state to few-layered one.

**Electronic supplementary material:**

The online version of this article (10.1007/s40820-020-0405-7) contains supplementary material, which is available to authorized users.

## Highlights

- A facile NH_4_^+^ method was proposed to prepare Sn nanocomplex pillared few-layered Ti_3_C_2_T_x_ MXene nanosheets.
- The MXene nanosheets showed excellent lithium-ion storage performances among MXene-based materials, which can maintain 1016 mAh g^−1^ after 1200 cycles at 2000 mA g^−1^ and deliver a stable capacity of 680 mAh g^−1^ at 5 A g^−1^.

**Electronic supplementary material:**

The online version of this article (10.1007/s40820-020-0405-7) contains supplementary material, which is available to authorized users.

## Graphical abstract

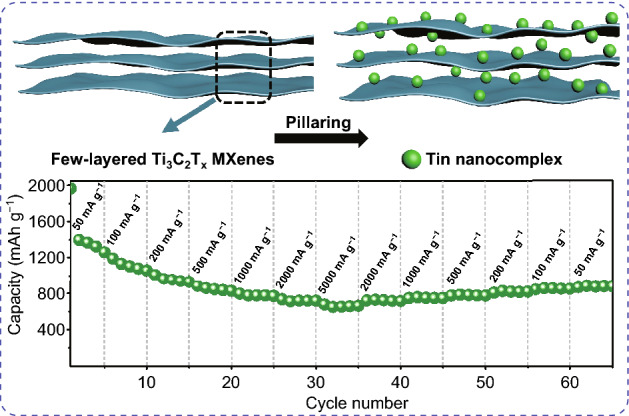

## Introduction

Two-dimensional (2D) materials have stimulated great research interest in the fields of energy storage, catalysis, sensor, semiconductor, etc, because of their novel physical and chemical properties [1–4]. MXenes are a new family of 2D transition metal carbides and nitrides discovered in 2011 [4, 5]. They can be prepared by selectively etching “A” atomic layers from 3D layered MAX phases (M_n+1_AX_n_, where *M* is an early transition metal, A is an IIIA or IVA group element, *n* = 1, 2, 3, and X is C and/or N) [6, 7]. The final chemical formula of MXenes can be denoted as M_n+1_X_n_T_x_; the meaning of each letter is the same as that of MAX phases (M_n+1_AX_n_), except for T_x_ representing terminated –OH, –F, and –O functional groups at surface [8, 9].

Although MXenes show promising application prospect in energy storage [10–12], the practical electrochemical performance of MXenes falls short of expectations [13]. This poor result is probably due to the blocking effect of surface anion and excessive accumulation of layers [14–16]. In order to improve the electrochemical performance of MXenes, many modification strategies have been carried out, including increasing the interlayer spacing by insertion of other agents [17–19], constructing 3D morphology [20, 21], and creating porous structure [22, 23]. The first modification strategy of enlarging interlayer spacing has been proved an effective method to improve the capacities of MXenes, and it is figuratively called “pillaring” technique. The pillaring agents could be small molecular cations [24], cationic surfactant [25], polymers [26], metal nanocomplex [27], and so on. Figure S1 shows the development history of pillaring technique in MXene-based electrode materials. In detail, the interesting pillared MXenes with active materials date back to 2016, and then, a series of works about pillared MXenes were reported from different perspectives, including various intercalation agents (CTAB [25], KOH [17]), active materials (Sn [25, 28, 29], Co [15], S [18]), and different battery systems [18, 25, 30]. Due to “pillar effect,” which means the interlayer spacing of MXene matrix was enlarged by active materials, providing more lithium storage interspace and endowing the pillared MXene nanocomposites excellent electrochemical performance [25, 28]. Unfortunately, up to now, all of these reports were blocked at the state of multilayered MXenes with thickness about 5 μm, corresponding to about 5000 layers calculated on the interlayer spacing of ~ 1 nm (Fig. S2) [13]. Particularly, due to serious restacking issues of few-layered MXene nanosheets [31, 32], there is no report about pillared few-layered MXene composites. It is well known that few-layered structure is considered to benefit the electrochemical properties of 2D materials [33–35]. Therefore, discovering effective method to prepare pillared few-layered MXene-based composites is of great significance; realization of pillared few-layered MXenes with active materials is still a big challenge to be taken.

Herein, for the first time, we synthesized atomic Sn nanocomplex pillared few-layered Ti_3_C_2_T_x_ (STCT) composites via pillaring technique with the assistance of NH_4_^+^ method and investigated its electrochemical properties as anodes for lithium-ion battery, effectively solving the restacking phenomenon of few-layered MXenes. Notably, benefiting from the few-layered nanosheet structure and pillared ultra-large interlayer spacing, the STCT composites exhibit significant improvement in electrochemical performance. It delivers outstanding cycling stability with a capacity retention of 1016 mAh g^−1^ after 1200 cycles and superb rate ability with a capacity retention of 680 mAh g^−1^ at 5 A g^−1^. The electrochemical performance is even superior than that of the pillared multilayered MXenes with low formula weights [15, 17], although the related theoretical study indicates that MXenes with low formula weights (such as V_2_C, Ti_2_C) show more promising prospect in energy storage than the ones with high formula weights [36]. The results strongly demonstrate the advantages of pillared few-layered MXenes in the field of energy storage.

## Experimental Section

### Synthesis Methods

#### Synthesis of Multilayered Ti_3_C_2_T_x_ MXenes

Commercial Ti_3_AlC_2_ powders (98% in purity) were purchased from Beijing Forsman Technology Company. The multilayered Ti_3_C_2_T_x_ MXene powders can be easily obtained according to previous report [28].

#### Synthesis of Few-layered Ti_3_C_2_T_x_ MXenes (NH^4+^ Method)

Multilayered Ti_3_C_2_T_x_ MXene powders (1 g) were immersed into 10 mL aqueous solution of tetramethylammonium hydroxide (25 wt%, TMAOH) and stirred at room temperature for 24 h. Organic molecules were intercalated into the interlayer of MXenes, and then, the intercalated multilayered Ti_3_C_2_T_x_ MXenes were collected by centrifugation and washed with deionized (DI) water for three times. The final precipitation was dispersed in 50 mL DI water for ultrasonic treatment at atmosphere of argon gas. After ultrasonication for 1 h, the supernatant was collected for subsequent usage via centrifugation at speed of 3500 r min^−1^ for 10 min, and the precipitation was again dispersed in 50 mL DI water for further ultrasonic treatment, and the above process was repeated for 4 times. Then, all the above-mentioned supernatants were collected together with nearly volume of 200 mL. In order to reduce water volume, shorten working time of freeze-drying process and avoid the restacking phenomenon of few-layered MXene nanosheets, 20 mL ammonia water (28%) or 25 mL ammonium bicarbonate aqueous solution (0.5 mol L^−1^) was added into the MXene supernatants, and an obvious electrostatic flocculation phenomenon can be observed. After resting for 1 h, the flocculation settles down automatically and can be collected. Few-layered Ti_3_C_2_T_x_ MXene powders can be obtained by freeze-drying of the flocculation and subsequent annealing treatment (180 °C, 6 h) with Ar atmosphere. Since the above-mentioned preparation process of few-layered MXene powders was first time proposed, we termed it as NH^4+^ method, which was first time put forward and can fundamentally solve restacking issues of few-layered MXene nanosheets. It should be noted that the other ammonium salts are also suitable for electrostatic flocculation process.

#### Synthesis of CTAB-Ti_3_C_2_T_x_ MXenes

The CTAB-Ti_3_C_2_T_x_ MXenes were prepared by treating Ti_3_C_2_T_x_ MXene powders in hexadecyl trimethyl ammonium bromide (CTAB) solutions as reported [28].

#### Synthesis of STCT Composites

As-prepared multilayered or few-layered Ti_3_C_2_T_x_ MXene powders (0.3 g) were immersed into 40 mL 0.2 wt% CTAB solution and stirred for 24 h at 40 °C, then 2.6 g ${\text{SnCl}}_{4} \cdot 5{\text{H}}_{2} {\text{O}}$ was added and mixed solution was stirred again for 24 h for ion-exchange process, and the final product of Sn nanocomplex pillared few-layered Ti_3_C_2_T_x_ (STCT)composites can be obtained after centrifugation (3500 r min^−1^, 10 min), freeze-drying, and annealing treatment in Ar atmosphere (180 °C, 2 h). Note that STCT composites are at few-layered state in this manuscript unless otherwise specified.

### Material Characterization

X-ray diffraction (XRD) measurements were taken by Shimadzu XRD 6000 with Cu Ka radiation in the range of 2*θ* = 3°–90°. AFM results were collected by Oxford Cypher S to detect the height of the nanosheets. The microstructure of the as-prepared samples was measured by field emission scanning electron microscopy (FESEM, Hitachi, SU8010) and transmission electron microscopy (TEM, FEI Ltd, Tecnai F20). X-ray photoelectron spectroscopy (XPS) analysis was carried out in Thermo Fisher 250XI to investigate chemical bonds of the samples. The discharged electrodes were washed with fresh dimethyl carbonate (DMC) solvent to remove surficial residuals before related characterization.

### Electrochemical Measurements

Coin type cells (2032) were assembled in argon-filled glove box using STCT composites as working electrodes and Li foil as the negative electrode. The electrodes were prepared with mixed STCT composites, super-P, and carboxyl methyl cellulose (CMC) to form slurry at the weight ratio of 7:1:2. Cyclic voltammogram (CV) curves at different scan rates between 0.01 and 3.0 V were obtained, and batteries were also analyzed by EIS measurements using Solartron 1470E Electrochemical Interface (Solartron Analytical, UK). Cycling performance and discharging/charging measurements were carried out on LAND battery test system.

## Results and Discussion

**Fig. 1:**
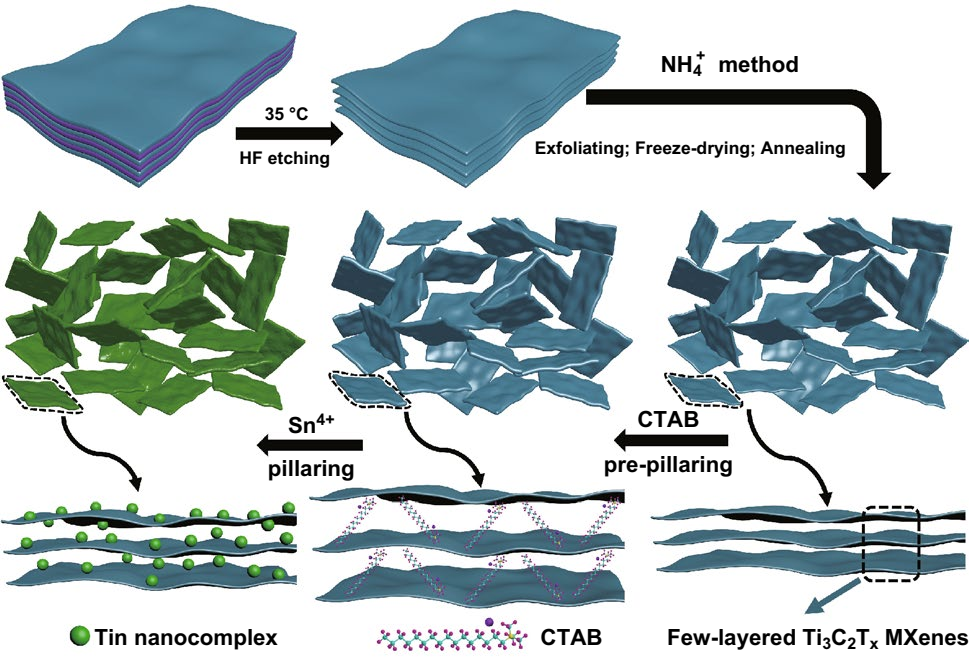
Schematic illustration of the preparation procedure of Sn nanocomplex pillared few-layered Ti_3_C_2_T_x_ (STCT) composites by etching, sonication, pre-pillaring, and pillaring processes

The preparation process of samples is illustrated in Fig. 1. Briefly, few-layered Ti_3_C_2_T_x_ MXene powders were prepared via NH_4_^+^ method, which can fundamentally solve the restacking issues of few-layered MXene nanosheets, and the detailed process can be seen in experimental section. Followed by CTAB pre-pillaring, ion-exchange insertion, and Sn^4+^ pillaring process, we finally obtained Sn nanocomplex pillared few-layered Ti_3_C_2_T_x_ (STCT) composites with few-layered structure. In contrast, multilayered Ti_3_C_2_T_x_ is obtained via HF etching without subsequent process (Fig. S3). Because of the delicate properties and structures, it is difficult to effectively prepare powders of few-layered MXenes from colloids after exfoliation process (Fig. S4). In this work, for the first time, we employed electrostatic flocculation of NH_4_^+^ method to prepare few-layered Ti_3_C_2_T_x_ nanosheet powders. Due to absorbed T_x_ groups, MXene nanosheet is negatively charged, which is also one of the basic properties of MXene colloids and consistent with the result of zeta potential test (Fig. S5) [37]. MXene nanosheets can be uniformly dispersed in colloidal state because of electrostatic repulsion among different nanosheets. The Ti_3_C_2_T_x_ nanosheets settle down after the addition NH_4_^+^ solution owing to destruction of electrostatic equilibrium state of the MXene nanosheets (Fig. S6a–e), leading to formation of flocculation [38], and the yield is considerable after freeze-drying of flocculation and annealing process (Fig. S7), ranging from 50 to 70% (Figs. S6f–g and S9).

**Fig. 2:**
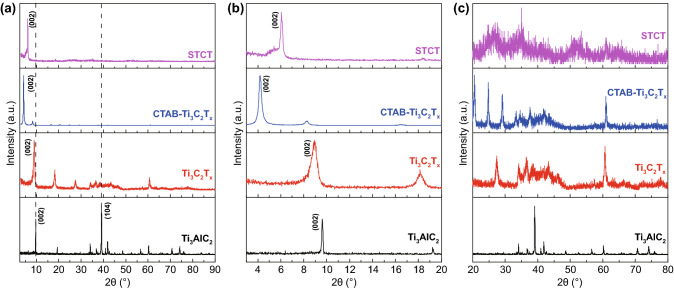
XRD patterns of Ti_3_AlC_2_, Ti_3_C_2_T_x_, CTAB-Ti_3_C_2_T_x_, and STCT composites at different diffraction angle ranges: **a** 2 theta from 3° to 90°, **b** 2 theta from 3° to 20°, and **c** 2 theta from 20° to 80°

The structure of materials in different steps is identified by XRD and exhibited in Figs. 2 and S8 with different diffraction angle ranges. There is an obvious shift in the main peak of (002), which explains well the change of interlayer spacing [25, 28, 38]. As shown in Fig. 2a, b, the disappearance of main peak (104) at 39° of Ti_3_AlC_2_ after HF etching suggests the complete etching of Al layer, and the left shift of peak (002) indicates enlarged interlayer spacing [6]. After immersing few-layered Ti_3_C_2_T_x_ in CTAB solution for hours, the main peak of Ti_3_C_2_T_x_ shifts dramatically to a lower angle, implying the high pillaring ability of cationic surfactant [25]. The intense diffraction peaks centered at 4° further confirm the successful intercalation of CTAB into the interlayers of Ti_3_C_2_T_x_ (Fig. 2c). Afterward, with the insertion of Sn nanocomplex via ion exchange based on Sn^4+^, the main peak of STCT composites shifts to a higher angle, which indicates the CTAB is replaced by Sn nanocomplex particles with small volume, resulting in interlayer spacing diminution [25]. As shown in Fig. 2c, the broad peaks corresponding to Sn nanocomplex are concealed by the intense diffraction peak of Ti_3_C_2_T_x_ MXenes.

**Fig. 3:**
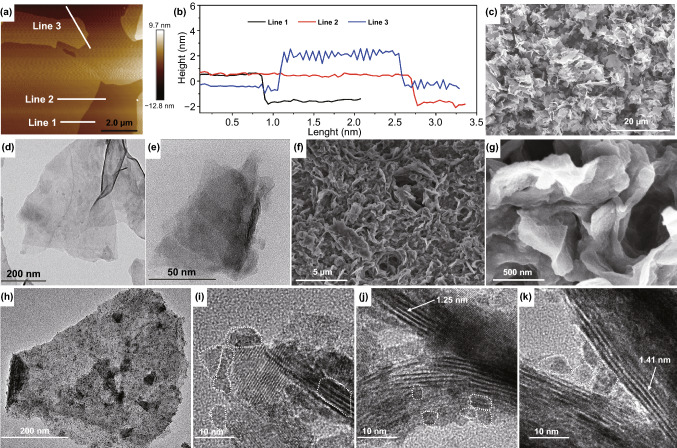
Characterization of few-layered Ti_3_C_2_T_x_ and STCT composites. **a, b** AFM images, **c** SEM image, and **d, e** TEM images of few-layered Ti_3_C_2_T_x_. **f, g** SEM images and **h–k** TEM images of STCT composites

As shown in Fig. 3a, b, the few-layered structure of Ti_3_C_2_T_x_ can be confirmed by AFM test. The scanned profile height of nanosheets indicates the number of layers of MXenes is about 2 or 3, demonstrating successful preparation of few-layered Ti_3_C_2_T_x_ MXene powders, which show obvious nanosheet structure with microscale size in *ab* plane. The uniform nanosheet morphology without agglomeration can be further testified by SEM images at different magnifications (Fig. S10 and Fig. 3c). The translucent state under transmission electron beam confirms the few-layered structure of Ti_3_C_2_T_x_ (Fig. 3d, e). The slight curl morphology indicates the flexibility of few-layered Ti_3_C_2_T_x_ MXene nanosheets. As the SEM images of STCT composites are shown in Fig. 3f, g, the lamellar structure remains in comparison with Ti_3_C_2_T_x_; nevertheless, the thickness of Ti_3_C_2_T_x_ slice increases slightly, suggesting the successful decoration of Sn nanocomplex. For better observation, the multilayered Ti_3_C_2_T_x_ is also pillared. As shown in Fig. S11, the interlayer space is padded by small particles, further confirming the Sn nanocomplex pillaring in Ti_3_C_2_T_x_ interlayers. TEM image in Fig. 3h clearly reveals the uniform distribution of Sn nanocomplex dots in the few-layered Ti_3_C_2_T_x_ matrix. The HRTEM images further exhibit the detailed distributed situation of Sn nanocomplex dots (Fig. 3i–k). The small Sn nanocomplex particles about 3–5 nm were anchored tightly at surface of Ti_3_C_2_T_x_, and ultrasmall atomic Sn nanocomplex dots were intercalated into the interlayers of Ti_3_C_2_T_x_ [15] or within the inner side of V-like construction between nanosheets. CTAB acts as pre-pillaring agent and surface-stabilizing agent, which effectively inhibits Sn nanocomplex particle growth and agglomeration [39]. The interlayer spacing of few-layered Ti_3_C_2_T_x_ is sizeable after CTAB pre-pillaring, which provides enough space for accommodating Sn nanocomplex dots (Fig. S12). The interlayer spacing further restricts grain growth and insures the ultrasmall Sn nanocomplex size.

**Fig. 4:**
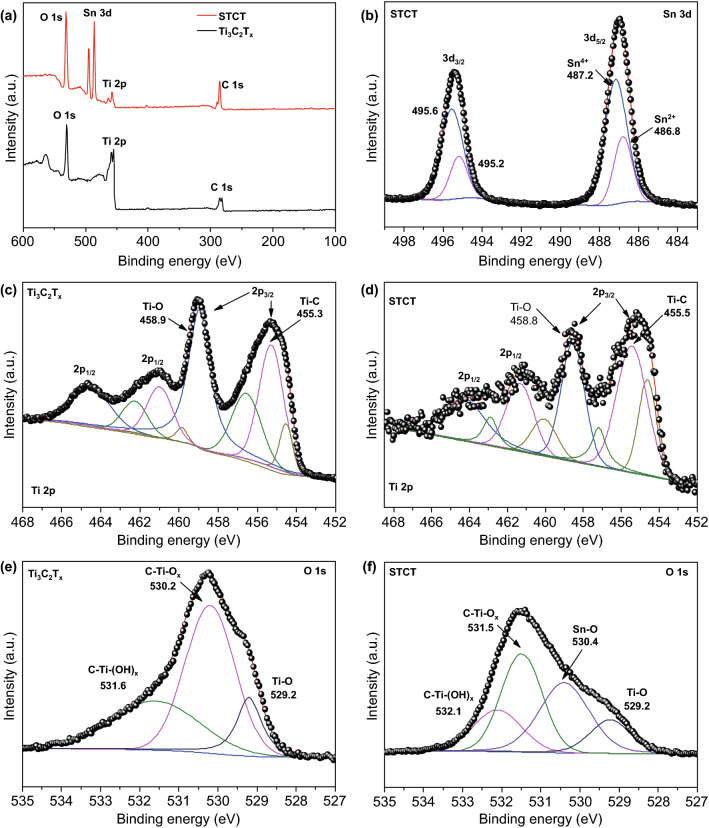
XPS spectra of **a** Ti_3_C_2_T_x_ and STCT composites, **b** high-resolution Sn 3d spectrum of STCT, **c–d** high-resolution Ti 2p spectra of Ti_3_C_2_T_x_ and STCT, and **e, f** high-resolution O 1 s spectra of Ti_3_C_2_T_x_ and STCT

The chemical bonding is collected by XPS. The full spectra comparison (Fig. 4a) of individual Ti_3_C_2_T_x_ and STCT composites further verifies the successful decoration of Sn nanocomplex. The detailed analysis shows that the Sn 3d peak consists of two primary peaks centered at 495.4 and 487.0 eV, attributed to the Sn 3d_3/2_ and 3d_5/2_, respectively. These two peaks can be deconvoluted into two pairs of peaks corresponding to Sn^2+^ and Sn^4+^, respectively, indicating the incomplete oxidation state of Sn nanocomplex [40, 41] (Fig. 4b). As shown in Fig. 4c, d, the Ti 2p peaks of Ti_3_C_2_T_x_ and STCT composites consist of four primary peaks, of which the two peaks with higher binding energy belong to Ti 2p_1/2_, while the lower two belong to Ti 2p_3/2_. These four peaks can be further split into eight peaks, respectively, corresponding to Ti^2+^, Ti^3+^, Ti–O, and Ti–C peaks [40, 42]. The O 1 s peak of Ti_3_C_2_T_x_ can be deconvoluted into three peaks, respectively, matched to the C–Ti–(OH)_x_, C–Ti–O_x_, and Ti–O peaks [42]. However, for the STCT composites, a peak corresponding to Sn–O bond (530.4 eV) emerges, indicating the bonding between Sn oxide particles with the terminating groups (such as –OH, –O) at the surface of Ti_3_C_2_T_x_ matrix [40]. In addition, slight shift of Ti 2p_3/2_ and O 1 s to higher binding energy also implies the forming of strong attraction between Sn nanocomplex and Ti_3_C_2_T_x_ matrix [28]. As shown in Fig. S13, the sharp decline of C–Ti peak intensity and emergence of C=O peak further indicate the formation of chemical binding between Sn nanocomplex particles and Ti_3_C_2_T_x_ matrix. The formation of interfacial Sn–O–Ti bonds can grant robust structure of the pillared composites, thereby assuring a stable electrochemical performance of STCT composites.

**Fig. 5:**
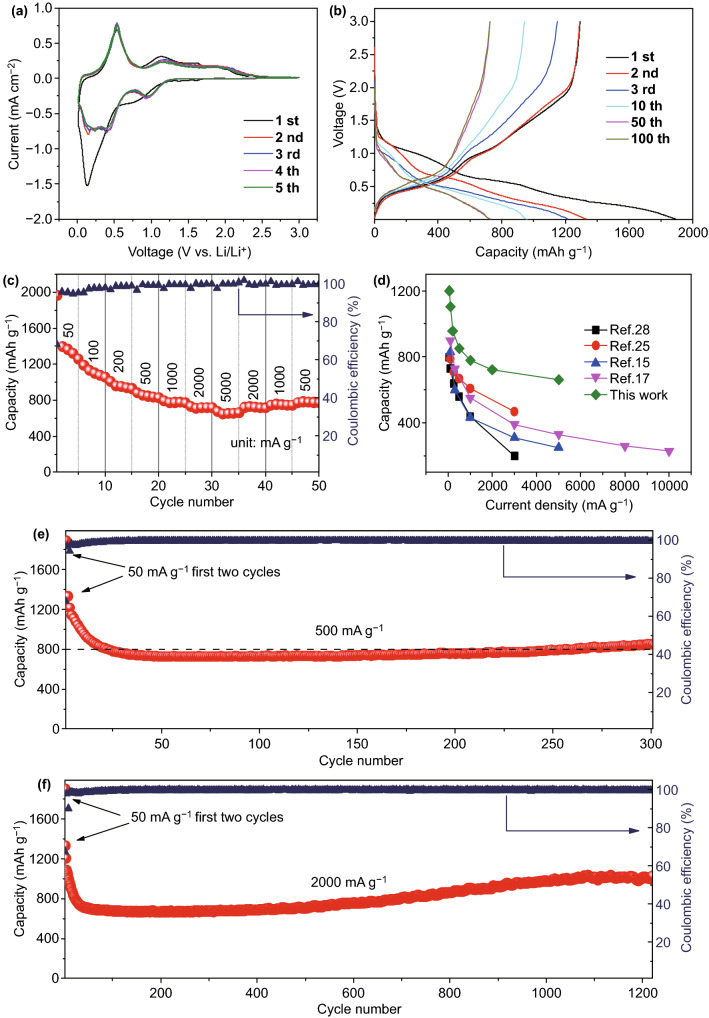
Electrochemical results of STCT composites: **a** CV curves at 0.1 mV s^−1^, **b** voltage–capacity curves of different cycles at 500 mA g^−1^, and **c** rate performance. **d** Rate performance comparison of different pillared MXene-based anode materials reported recently (just focus on closely related pillared MXene-based composites), **e** cycle performance at 500 mA g^−1^, and **f** long-term performance at a high current density of 2000 mA g^−1^

In order to evaluate electrochemical performance of STCT composites, we assembled 2032 type coin cell using lithium metal foil as counter electrode. As shown in Fig. 5a, the CV curves of STCT composites were measured between 0.01 and 3 V. The cathodic and anodic current response can be attributed to the electrochemical reactions between lithium ions and Sn nanocomplex as well as Ti_3_C_2_T_x_ matrix. The redox peak current positions are consistent with previous reports [40, 43, 44]. In the first cathodic scanning process, a broad hump centered around 0.8 V is attributed to the formation of solid electrolyte interface (SEI) layers and the reduction reaction of Sn nanocomplex with Li^+^ to form Sn and Li_2_O; the latter reaction is reversible and reflects a stable peak at 0.92 V in subsequent cycles [44–46]. The dominant peak below 0.5 V owes to electrochemical lithium storage by forming Li_x_Sn and trapping of Li^+^ within the Ti_3_C_2_T_x_ matrix, which are partially reversible and mainly responsible for sharp capacity decay at the first cycle [25, 40]. The reduction peaks are greatly different at subsequent scans compared with the first scan, suggesting the dynamic enhancement after the initial lithiation/delithiation activation combined with partially reversible reaction [28, 47]. In the anodic scanning, the dominant peak at approximately 0.54 V belongs to dealloying reaction of Li_x_Sn. Remarkably, two minor peaks are detected around 1.2 and 1.9 V, which are related to reversible conversion reaction of Sn to SnO and SnO to SnO_2_, respectively [48, 49]. The good overlap of CV curves after the initial scan demonstrates the good reversibility and robustness of the STCT composite electrodes at subsequent cycles [28, 50].

The galvanostatic charging/discharging curves of STCT composites at different cycles are presented in Fig. 5b. The charging/discharging platforms have one-to-one corresponding relationship with the redox peaks in CV curves. The discharge and charge capacities of the first cycle are 1892.4 and 1291.7 mAh g^−1^, respectively, with an initial coulombic efficiency of 68.3%. This value is relatively high among the Ti_3_C_2_T_x_-based electrode materials [18, 21, 25, 40]. The irreversible capacity of the first cycle comes from unavoidable formation of SEI layer, irreversible conversion reaction of partial Sn nanocomplex, and irreversible capture of Li^+^ in layered Ti_3_C_2_T_x_ matrix [28, 40]. The coulombic efficiency rapidly climbs to 97.2% at the second cycle and remains stable above 99.7% during subsequent cycles. There is a descent in the initial 30 cycles, which may result from irreversible lithiation of Ti_3_C_2_T_x_ MXene matrix (Fig. 5e, f) [50, 51]. The capacity stabilizes at about 745 mAh g^−1^ at 500 mA g^−1^ and no capacity decay in subsequent cycles, indicating the excellent electrochemical performance because of stable structure of STCT composites, which can work steadily over 1000 cycles with obvious electrochemical platforms (Figs. S14 and S19). SEM and TEM images of STCT electrodes after cycles are shown in Fig. S15, the cycled STCT electrodes remain stable structure, and no fissures can be observed at surface. Furthermore, the Sn nanocomplex particles are still dispersed uniformly at Ti_3_C_2_T_x_ matrix after cycles, without agglomeration or pulverization (Fig. S16). The capacities of raw Ti_3_AlC_2_ material and multilayered Ti_3_C_2_T_x_ are quite low under the same test conditions, only delivering about 60 and 121 mAh g^−1^ (Fig. S17). The multilayered STCT composites deliver a reversible capacity of 367.4 mAh g^−1^ after 100 cycles at 500 mA g^−1^ (Fig. S18), which is about triple of the capacity provided by individual multilayered Ti_3_C_2_T_x_ without pillaring treatment. The result further demonstrates the significant effect of pillaring technique. An upward trend of capacity is observed upon cycling, especially in the long-term cycling at 2000 mA g^−1^. The capacity increases from 761.4 mAh g^−1^ at the 600th cycle to 1010.2 mAh g^−1^ at the 1075th cycle, giving a high capacity retention of 142% compared with the 40th cycle (Fig. S19). The uplift of capacity can be ascribed to the increased interlayer spacings of Ti_3_C_2_T_x_ matrix [51–53], which are propped by the volume expansion of Sn nanocomplex during lithiation and persistent shuttle of ions and electrons in the interlayers, leading to continuous enhancement of lithium storage capacity of the composites upon cycling [25]. It is found that a higher current density can induce earlier uptrend of capacity due to more violent ion and electron flows, leading to faster expansion of the interlayer spacing (Fig. S20 and Table S1).

The rate performance of STCT electrode can be measured at different current densities from 50 to 5000 mA g^−1^ (Figs. 5c and S21). As the current density increases, the capacity decreases progressively. The stable discharging capacities at 500 and 5000 mAh g^−1^ are 881.5 and 662.2 mAh g^−1^, respectively, indicating that the capacity remains up to 75.1% even the current density increases 10 times from 500 to 5000 mA g^−1^. As the current density returns to 500 mA g^−1^, the capacity recovers to 793.3 mAh g^−1^, suggesting the good reversibility of the electrode (Fig. S21a–c). The electrode can still cycle steadily at 500 mA g^−1^ after rate test, indicating the stable structure during high-rate test (Fig. S21b). The outstanding rate performance mainly benefits from the ultrathin Ti_3_C_2_T_x_ layers, the ultrafine Sn nanocomplex particles, and the “pillar effect” that caused increasing interlayer spacing of Ti_3_C_2_T_x_ nanosheet matrix [28, 41, 54]. This rate capability and corresponding specific capacities in LIBs are much better than previous reports about pillared MXenes due to usage of few-layered MXenes without restacking phenomenon (Figs. 5d and S22) [15, 17, 25, 28], further demonstrating the effectiveness of NH^4+^ method and advantages of pillared few-layered MXenes.

**Fig. 6:**
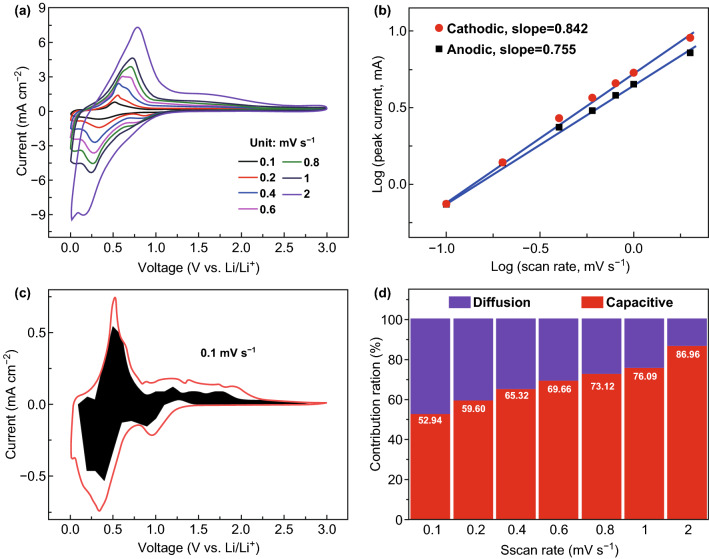
**a** CV curves of STCT electrodes at different scan rates from 0.1 to 2 mV s^−1^. **b** Plots for log (*i*) versus log (*v*), where *i* is the peak current and *v* is the scan rate. **c** Capacitive contribution proportion compared with the total current response at 0.1 mA s^−1^. **d** Bar charts of the capacitive contribution proportion at different scan rates (from 0.1 to 2 mV s^−1^)

In order to investigate reason for the excellent lithium storage of STCT composites, we analyzed the electrochemical kinetic mechanism by testing CV curves at various sweep speeds ranging from 0.1 to 2 mV s^−1^ (Fig. 6). As shown in Figs. 6a and S23a, b, a regular negative shift for the cathodic peaks and positive shift for the anodic peaks occur because of the polarization enhancement as the sweep speed increases [55]. The relationship between the peak current (*i*) and the scan rate (*v*) obeys the power law (Eqs. 1 and 2) [56]:

1 $$i = av^{b}$$

2 $${ \log } i = b{ \log } v + { \log } a$$

where *a* and *b* are adjustable constants. The *b* value can reflect the dominant factor of the electrochemical process. When the *b* value is close to 1.0, the electrochemical reaction is mainly controlled by the surface capacitive effect, while when the *b* value is close to 0.5, a solid-state diffusion-controlled process should be dominant [57]. As shown in Fig. 6b, the *b* value can be determined through calculating the slope of the log(*v*)–log(*i*) plots. The *b* values for the cathodic and anodic processes are estimated to be 0.842 and 0.755 (Fig. S23c, d), respectively, indicating a more favored surface-dominated lithium storage mechanism of STCT electrode [58].

The capacity contributions from surface-dominated and diffusion-dominated processes can be further quantitatively analyzed through the analysis approach established by Dunn et al. [59]. The current response of CV scan at a potential (*V*) varies with the scan rate (*v*) and obeys Eq. 3:

3 $$i\left( V \right) = k_{1} v + k_{2} v^{1/2}$$

$k_{1} v$ and $k_{2} v^{1/2}$, respectively, represent the capacitive and diffusion-dominated processes [60] where $k_{1}$ and $k_{2}$ are adjustable constants. Figure 6c shows the typical quantitative separation of capacitive current response compared with the total current response at a scan rate of 0.1 mV s^−1^. The capacitive effect contribution (black-shaded area) composes about 52.94% of the total capacity (red line surrounded area). As the scan rate rises from 0.1 to 2 mV s^−1^, the capacitive contribution proportion gradually increases from 52.94 to 86.96%. The analysis result manifests the important role of capacitive lithium storage process, especially at high current densities. The high proportion of surface-controlled process implies the short ion transport path and efficient charge transfer kinetics, thereby pledging the excellent rate capability and superior cycling stability. The electrochemical kinetic result well explains the excellent electrochemical performance of STCT composites and further testifies the superior kinetic characteristics of pillared few-layered Ti_3_C_2_T_x_ MXenes.

Based on the characterization and electrochemical test results, benefited from “pillar effect,” the pillared few-layered Ti_3_C_2_T_x_ nanosheets are ideal carrier for Sn nanocomplex; STCT composites were synthesized successfully and show outstanding electrochemical performance, which can be attributed to the following reasons: (1) The pillared few-layered Ti_3_C_2_T_x_ matrix with enlarged interlayer spacing behaves as efficient lithium storage container [28], which can also act as 2D conductive network to enhance the electron transfer and Li^+^ transport [61, 62]. (2) The Ti_3_C_2_T_x_ can effectively confine the Sn nanocomplex particles within interlayer and work as a flexible skeleton to keep the structural stability of the composites during cycling process. (3) In addition, the ultrasmall Sn nanocomplex dots further reinforce the kinetics properties [63]. Therefore, the conversion reaction of Sn oxide is partially reversible in STCT composites. The reversible utilization of Li_2_O can not only increase the specific capacity of the electrode, but also improve the coulombic efficiency [64, 65]. (4) The atomic Sn nanocomplex particles are anchored into the few-layered Ti_3_C_2_T_x_ matrix through electrostatic or chemical adsorption via formation of Sn–O–Ti bonding; the grain growth and aggregation of the particles are thoroughly restrained. The Sn nanocomplex can act as ideal pillaring agent to significantly improve the capacity of Ti_3_C_2_T_x_, because the drastic volume fluctuation during lithiation/delithiation will further pry the interlayer of Ti_3_C_2_T_x_ and acquire more lithium storage interspace, leading to fascinating electrochemical performance, especially rate performance. As far as we know, the STCT composites exhibit the best electrochemical performance among tin-based materials/MXene composites reported so far, and one of the best among the MXene-based materials (Table S2). Pillared few-layered MXenes can provide enlightenment for the insightful development of pillared MXenes, which open a new path for the application of MXenes due to larger interlayer spacing. In the future, it is believed the MXenes of *n* = 3 in chemical formula (M_n+1_C_n_T_x_) are more attractive because of intrinsic larger interlayer spacing (1.4 nm) than that of *n* = 2 (1 nm) or *n* = 1 (0.7 nm) [15, 25, 66–69].

## Conclusions

In summary, we design a facile NH^4+^ method to fundamentally solve the restacking phenomenon of few-layered MXenes, which is important for further study of various MXenes. Besides, partial atomic Sn nanocomplex pillared few-layered Ti_3_C_2_T_x_ composite (STCT) was fabricated by CTAB pre-pillaring and Sn nanocomplex pillaring process. The pillared few-layered structure is deemed responsible for the superior electrochemical performance of the as-prepared MXene-based material. The novel few-layered structure will not only facilitate the full utilization of Ti_3_C_2_T_x_ in lithium storage, but also improves the dynamic properties of decorated Sn nanocomplex. The ingenious combination of Ti_3_C_2_T_x_ and Sn nanocomplex can generate “pillar effect.” On the one hand, the highly conductive Ti_3_C_2_T_x_ can effectually restrain Sn nanocomplex particle growth and agglomeration, thereby ensuring the high use ratio of Sn nanocomplex and activating reversible conversion reaction of Sn back to Sn oxide. On the other hand, the large volume expansion of Sn nanocomplex during charging/discharging processes will further open the interlayers of Ti_3_C_2_T_x_ and allow more Li^+^ storage; as a result, Sn nanocomplex pillared few-layered Ti_3_C_2_T_x_ composite exhibits outstanding electrochemical performance: It delivers a stable discharging capacity about 800 mAh g^−1^ at 500 mA g^−1^ and maintains 1016 mAh g^−1^ after 1200 cycles at 2000 mA g^−1^. A high capacity retention of 680 mAh g^−1^ is also obtained at 5 A g^−1^, suggesting remarkable rate ability of STCT composites. Our work demonstrates the pillared few-layered MXene nanosheets are ideal matrix for high capacity anode materials with large volume expansion. We believe this work can provide enlightenment for the modification of other energy storage materials.

## Electronic supplementary material

Below is the link to the electronic supplementary material.

Supplementary material 1 (PDF 2460 kb)
