# Supplementary material for: Partial Atomic Tin Nanocomplex Pillared Few-Layered Ti3C2Tx MXenes for Superior Lithium-Ion Storage
Source: Nanomicro Lett. 2020 Mar 25;12:78. doi: 10.1007/s40820-020-0405-7 (PMC7770861; doi:10.1007/s40820-020-0405-7)
Supplement: Supplementary file 1 — Supplementary material 1 (PDF 2460 kb) [file 40820_2020_405_MOESM1_ESM.pdf]

Supporting Information for

Partial Atomic Tin Nanocomplex Pillared Few-layered $Ti_3C_2T_x$ MXenes for Superior Lithium-Ion Storage

Shunlong Zhang[1,⊥], Hangjun Ying [1,⊥], Bin Yuan[2,3], Renzong Hu[2,3], Wei-Qiang Han [1,*]

[1] School of Materials Science and Engineering, Zhejiang University, Hangzhou 310027, People's Republic of China

[2] School of Materials Science and Engineering, South China University of Technology, Guangzhou 510641, People's Republic of China

[3] Key Laboratory of Advanced Energy Storage Materials of Guangdong Province, Guangzhou 510641, People's Republic of China

⊥ Shunlong Zhang and Hangjun Ying contributed equally to this work

***Corresponding Author:** hanwq@zju.edu.cn(Wei-Qiang Han)

Supplementary Figures and Tables

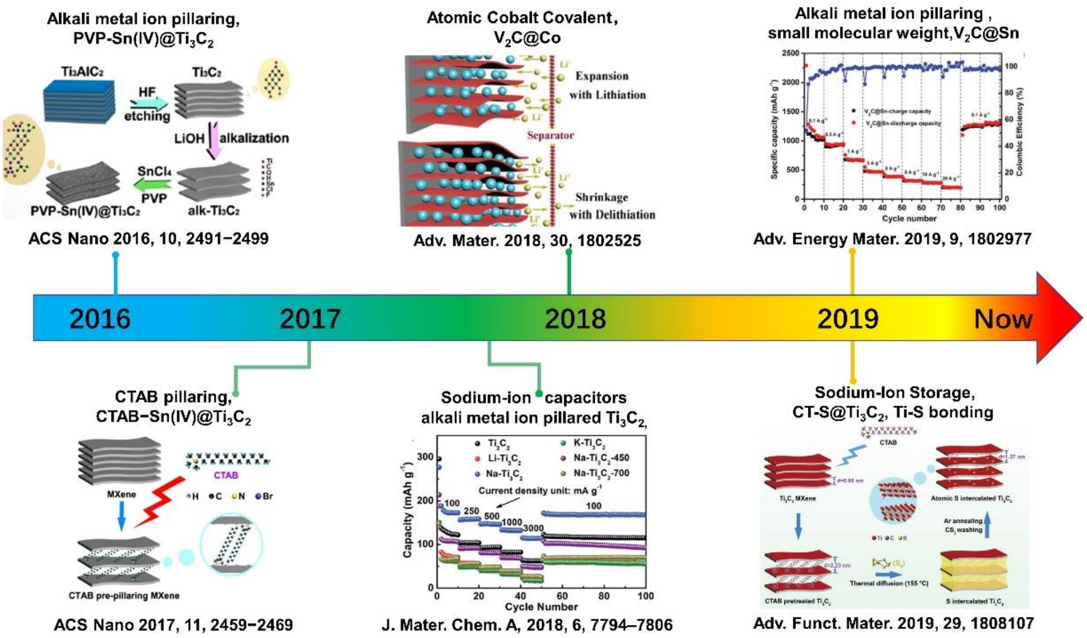

Fig. S1. Schematic development history of pillared MXenes-based composites accompanied with increased interlayer spacing.

Despite a considerable work about pillared MXenes-based materials were reported since 2016. Unfortunately, up to now, all these reports were blocked in the state of multi-layered MXenes due to restacking of few-layered MXenes. In this paper, through NH^{4+} method, the restacking phenomenon of few-layered MXenes was solved fundamentally.

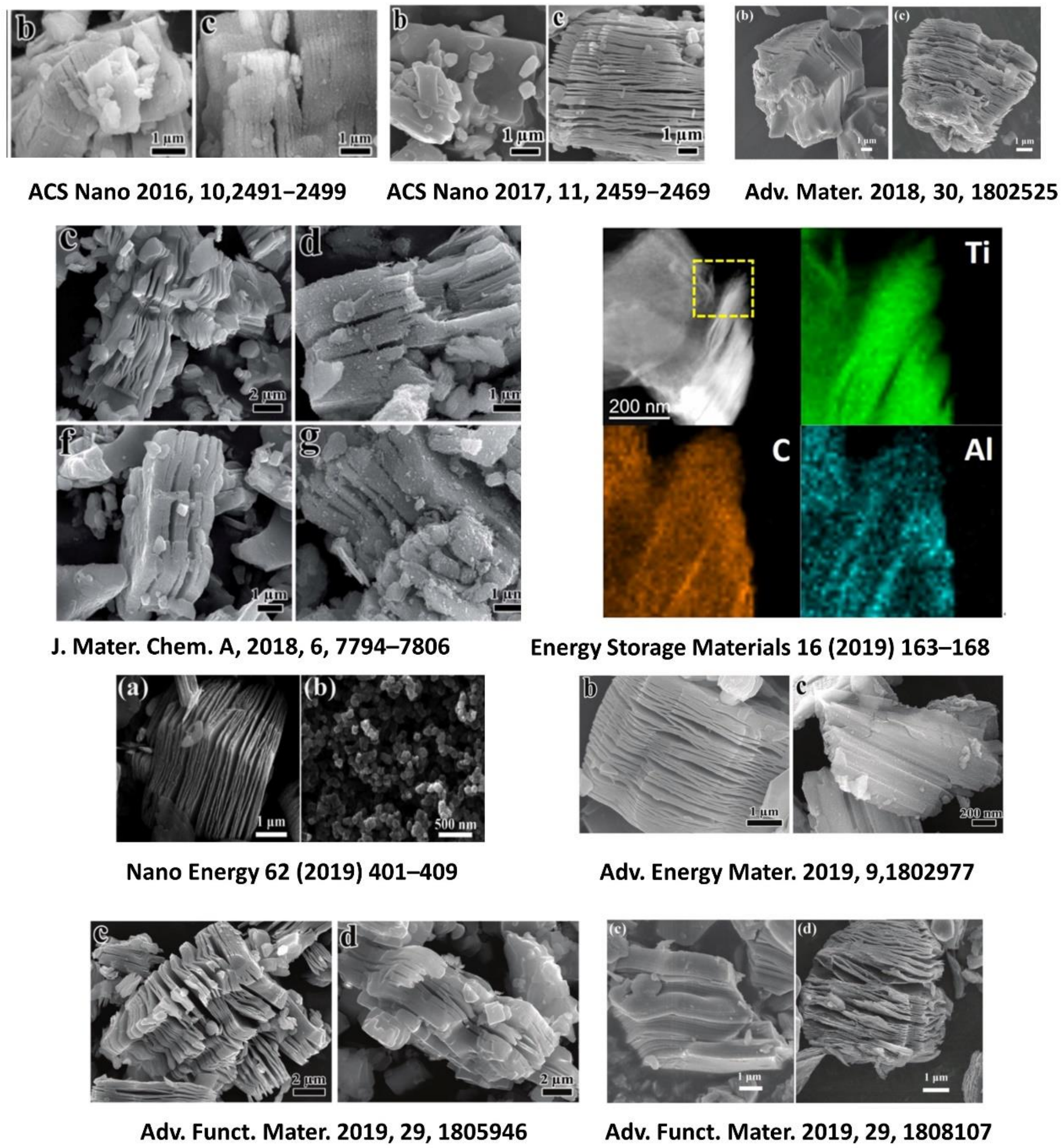

Fig. S2. Screenshot SEM images of pillared multi-layered $Ti_3C_2T_x$ MXenes in the previous reports.

We can clearly conclude that these reports were blocked in the state of multi-layered MXenes with thickness about 5 um, corresponding to 5000 layers calculated on the interlayer spacing of ~1nm, there are no report about pillared few-layered MXenes composites (below 5 layers) because of unsolved restacking phenomenon of few-layered MXenes.

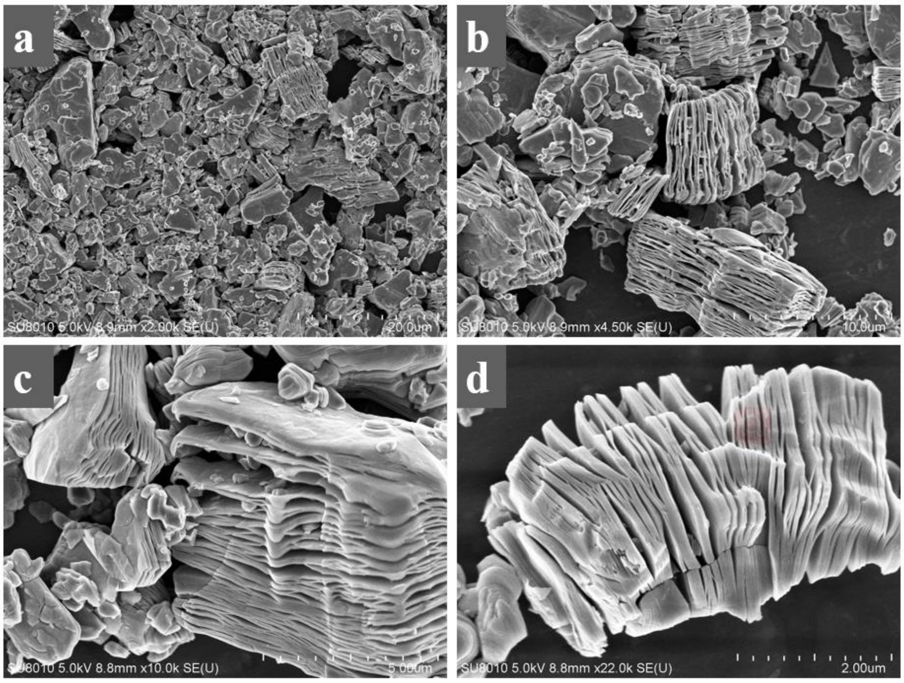

Fig. S3. (a)-(d) SEM images of multi-layered $Ti_3C_2T_x$ MXenes after etching from Ti_3AlC_2 MAX phases.

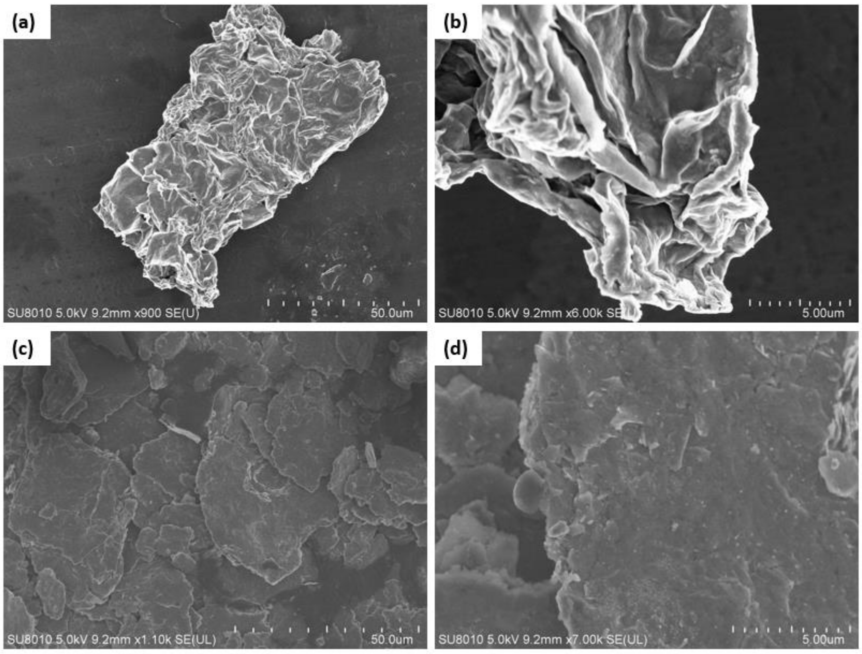

Fig. S4. SEM images of few-layered $Ti_3C_2T_x$ MXenes prepared through common methods. (a)-(b) centrifugation at high speed and freezing-dry method. (c)-(d) direct heating drying method.

The SEM images at low magnification times clearly show serious agglomeration of few-layered $Ti_3C_2T_x$ MXenes prepared via common methods, demonstrating that it's difficult to prepare powders of few-layered MXenes from solutions without agglomeration phenomenon.

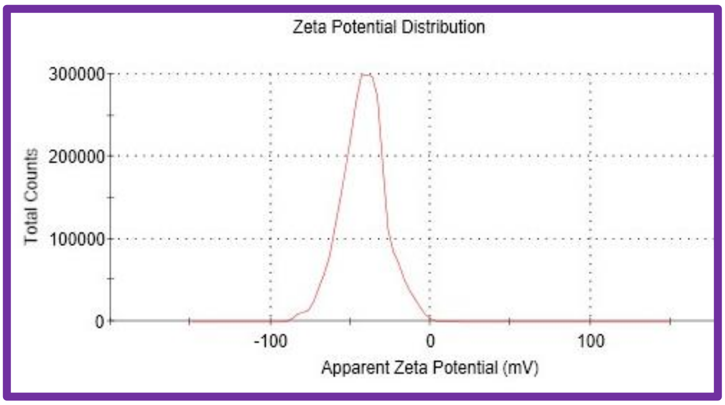

Fig. S5. Zeta potential of the $Ti_3C_2T_x$ MXenes colloids (-41.6mV)

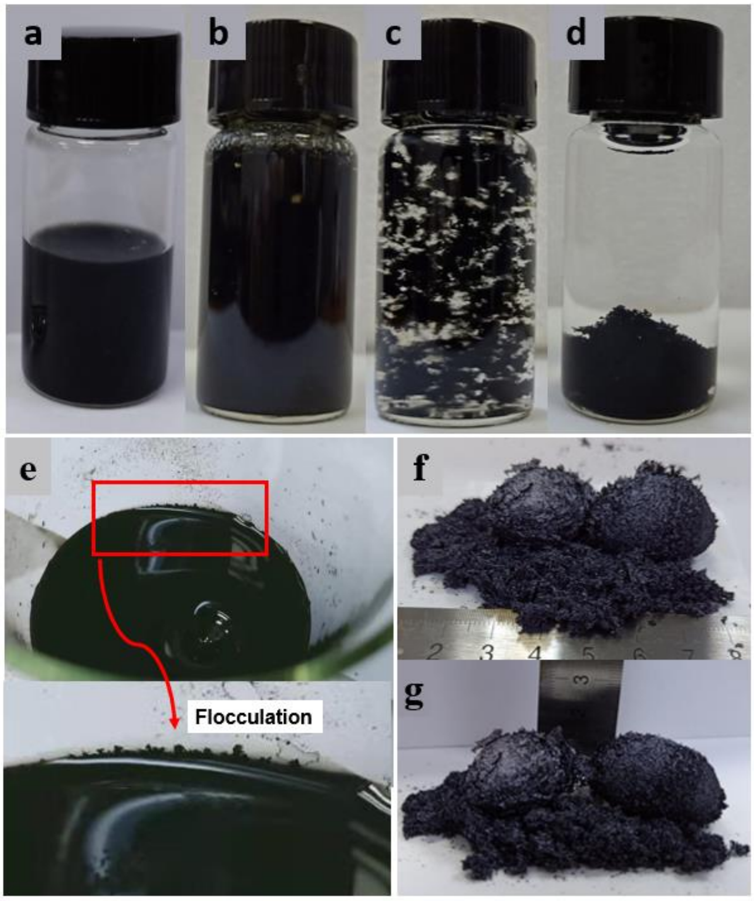

Fig. S6. Photographs of related solutions and final product. (a) Few-layered MXenes aqueous solution after exfoliation and sonication process. (b)The fresh state of few-layered MXenes solution with addition of NH_4^+ (ammonia or ammonium salts). (c) After 5 minutes of resting time. (d) After 1 hour of resting time. (e) magnified flocculation with stirring after adding NH_4^+ (ammonia or ammonium salts) process at beaker. (f)-(g) photographs of final few-layered $Ti_3C_2T_x$ powders after automatically electrostatic precipitation, complete flocculation, and freeze-drying process, showing fluffy and soft state overall.

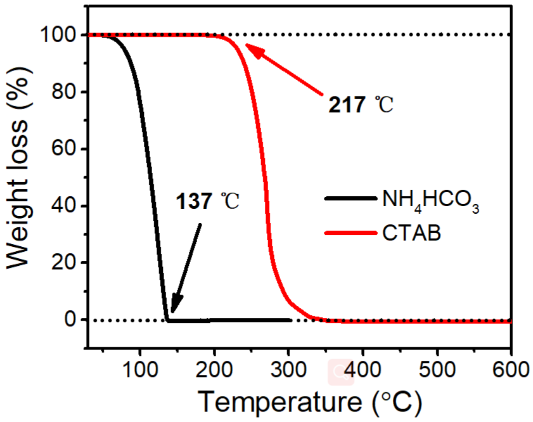

Fig. S7. TG analysis of ammonium bicarbonate (NH_4HCO_3) and hexadecyl trimethyl ammonium bromide (CTAB) powders in Ar atmosphere.

Ammonium bicarbonate was volatilized completely before 137 °C and CTAB was stable before 217 °C.

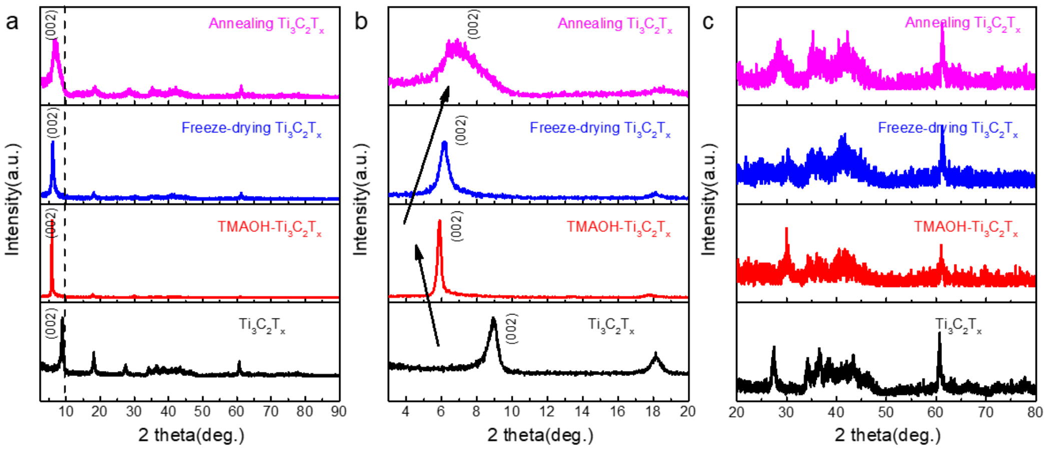

Fig. S8. XRD patterns of $Ti_3C_2T_x$, TMAOH-$Ti_3C_2T_x$, Freeze-drying $Ti_3C_2T_x$, Annealing $Ti_3C_2T_x$ powders at different diffraction angle ranges. (a) 2 theta from 3° to 90°. (b) 2 theta from 3° to 20°. (c) 2 theta from 20° to 80°.

There is a significant shift in the main peak of (002), which explains well the change of interlayer spacing. TMAOH organic molecules treating process enlarged the interlayer spacing of $Ti_3C_2T_x$ MXenes, after freeze-drying and annealing process, the interlayer spacing reduced because of removing molecules or ions from the interlayer. However, the interlayer spacing of few-layered $Ti_3C_2T_x$ MXenes is still larger than that of multi-layered $Ti_3C_2T_x$ MXenes due to aforementioned treatment.

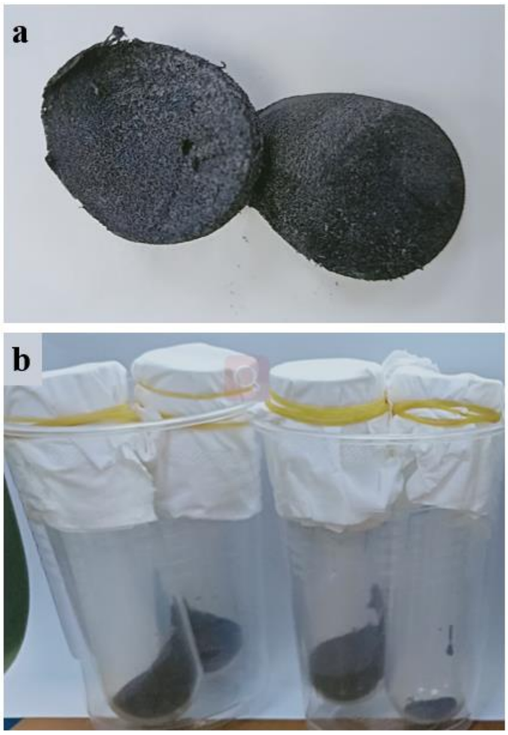

Fig. S9. photographs of final few-layered $Ti_3C_2T_x$ after low speed centrifugation and freeze-drying process, showing slightly compact in comparision with automatically electrostatic precipitation and complete flocculation.

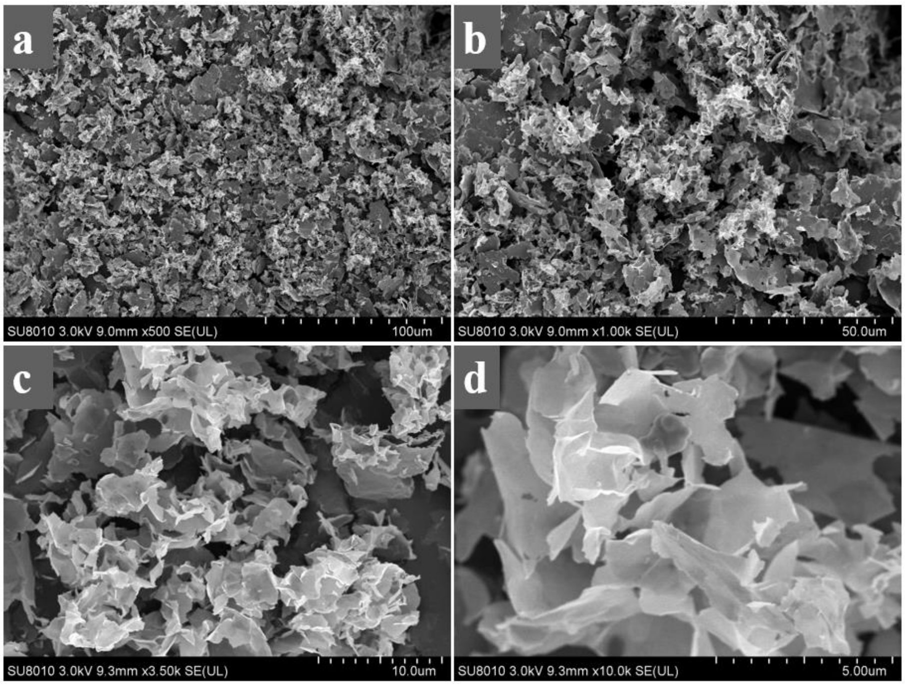

Fig. S10. (a)-(d) SEM images of few-layered $Ti_3C_2T_x$ MXenes. This is the first time to give SEM images of $Ti_3C_2T_x$ MXenes at a lower magnification of 500 times. The results show no aggregations or bulks of $Ti_3C_2T_x$ MXenes nanosheets existed in the powders, demonstrating the successful preparation of few-layered $Ti_3C_2T_x$ nanosheets through NH_4^+ method.

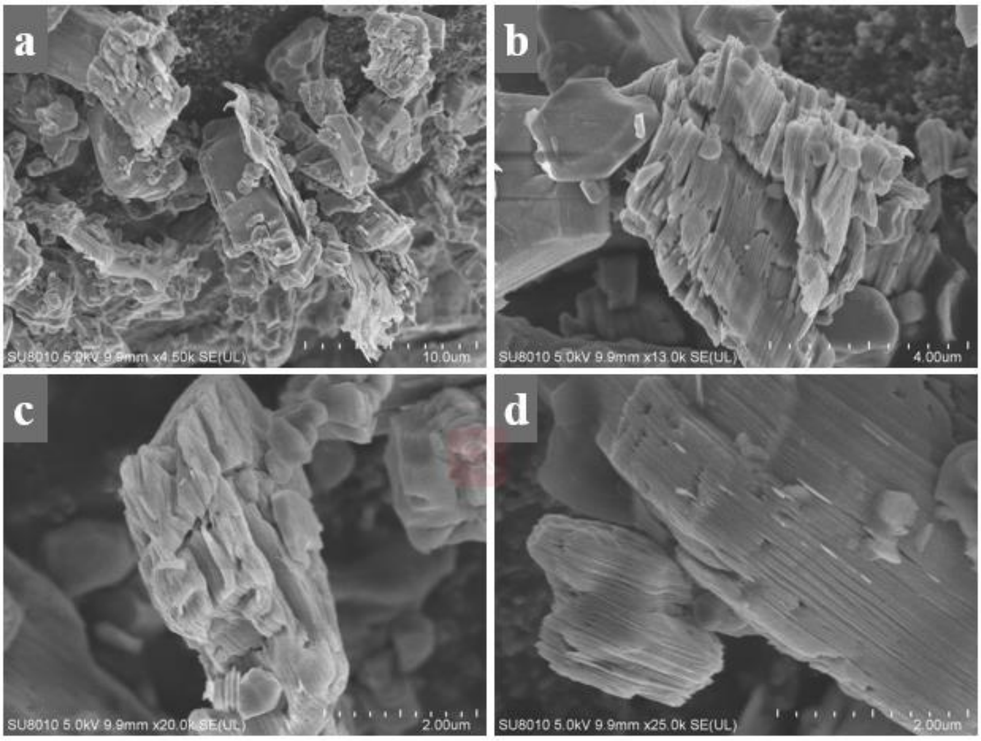

Fig. S11. SEM image of multi-layered STCT composites with CTAB prepillaring process.

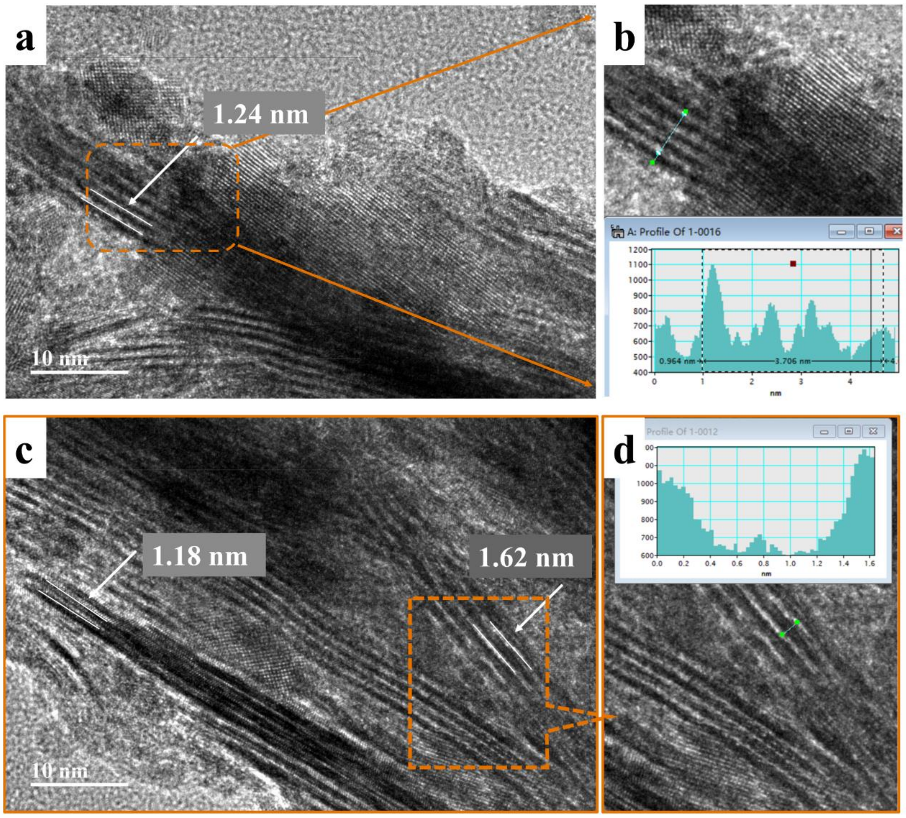

Fig. S12. TEM image of few-layered STCT composites with CTAB prepillaring process.

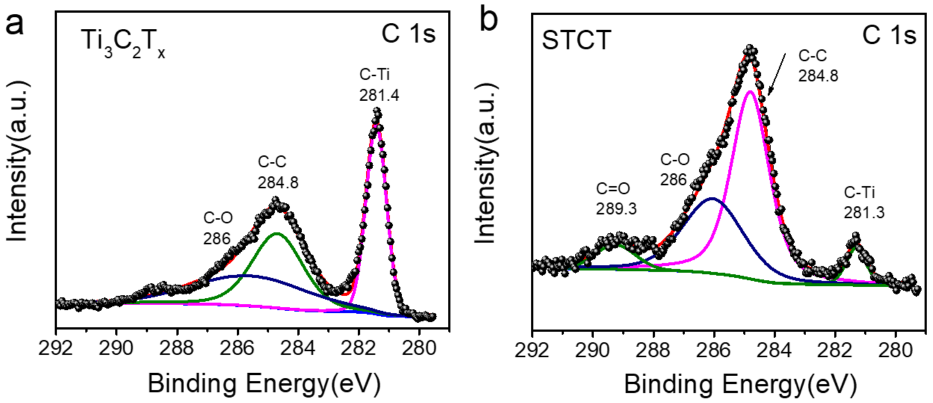

Fig. S13. X-ray photoelectron spectroscopy (XPS) study. (a) High resolution C 1s spectrum of the $Ti_3C_2T_x$ MXenes. (b) High resolution C 1s spectrum of the STCT composites.

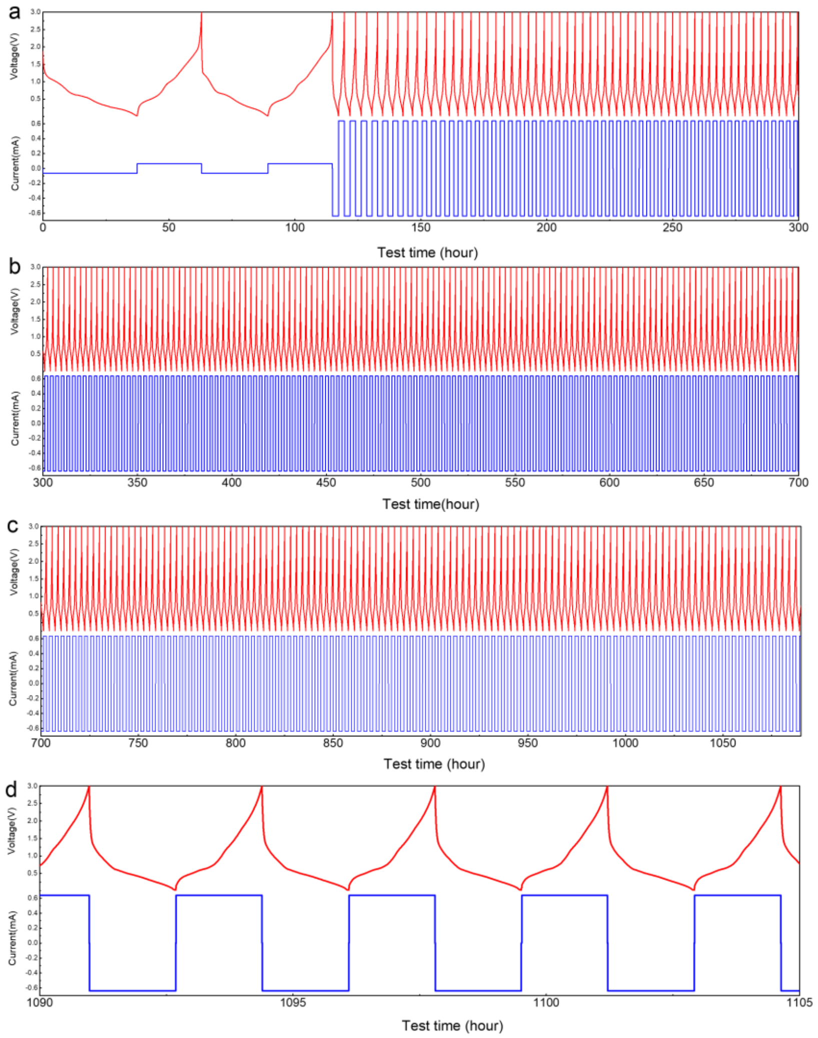

Fig. S14. Discharging and charging voltage/current profiles of STCT composites at 500 mA/g in different testing time. (a) initial 300 h, (b) 300 h to 700 h, (c) 700 h to 1090 h, (d) 1090 h to 1105 h.

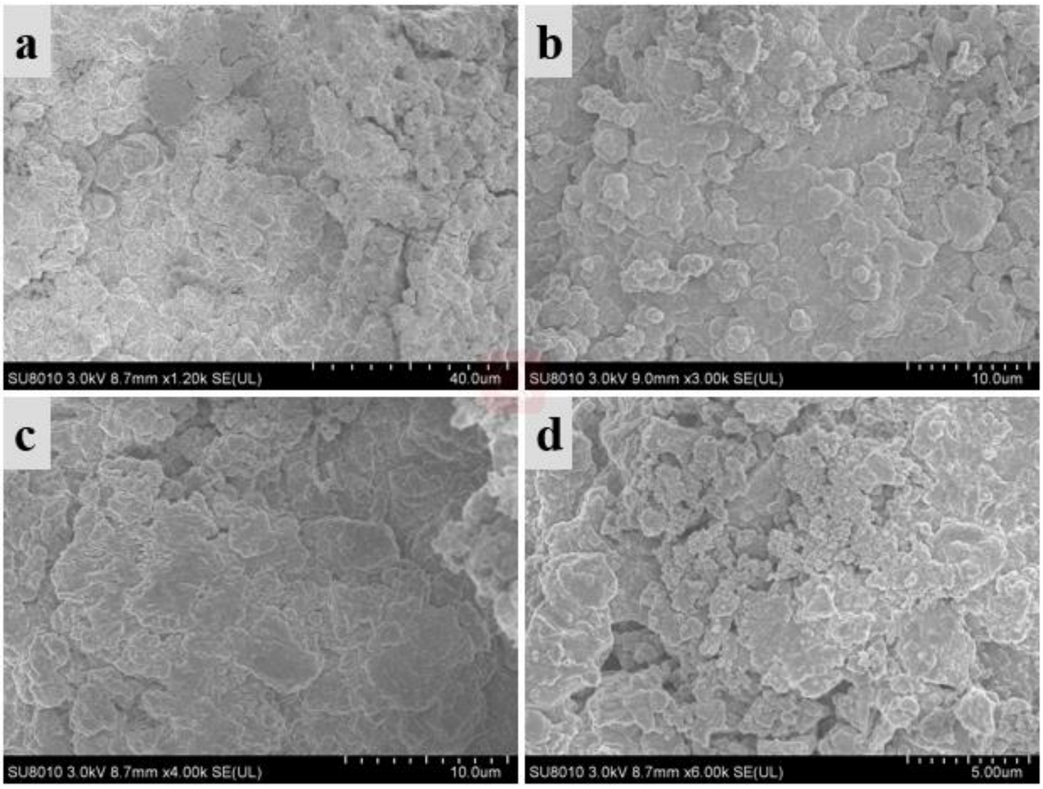

Fig. S15. SEM images of few-layered STCT electrodes after 1000 cycles at a current density of 2000mA/g.

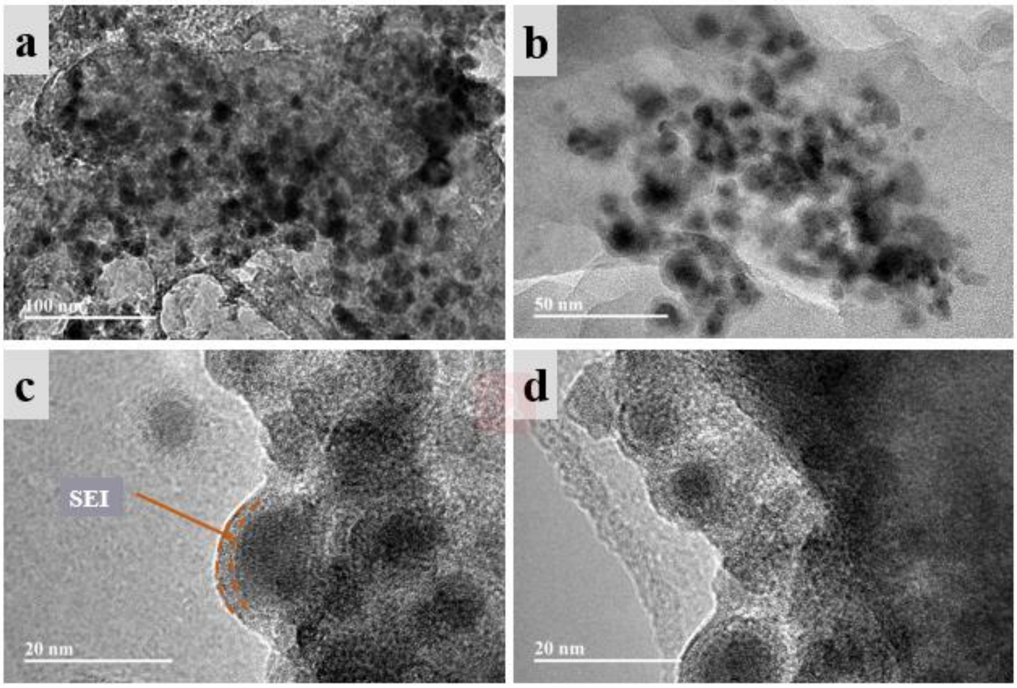

Fig. S16. TEM images of few-layered STCT composites after 1000 cycles at a current density of 2000mA/g.the results show no agglomeration tendency of active materials and indicate the formation of SEI film.

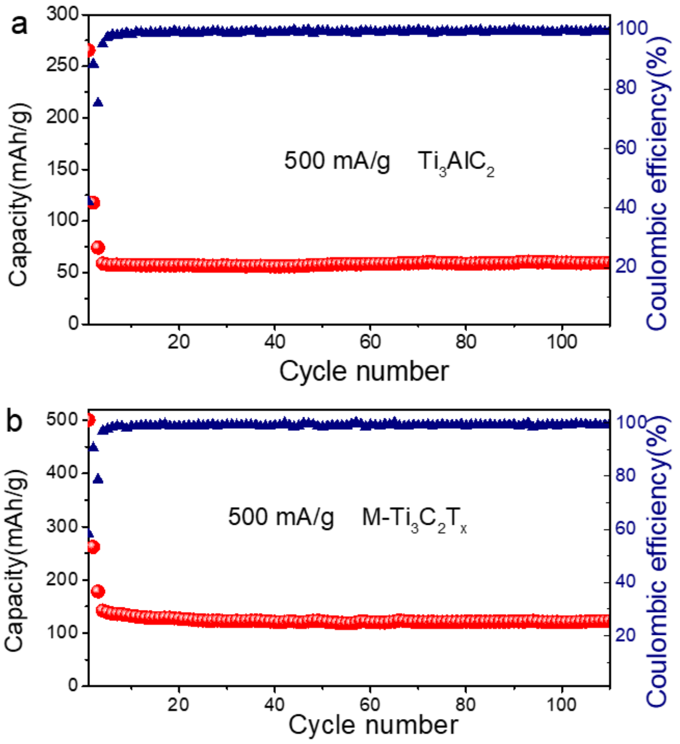

Fig. S17. Electrochemical performance of Ti_3AlC_2 MAX phases and multi-layered $Ti_3C_2T_x$ MXenes.

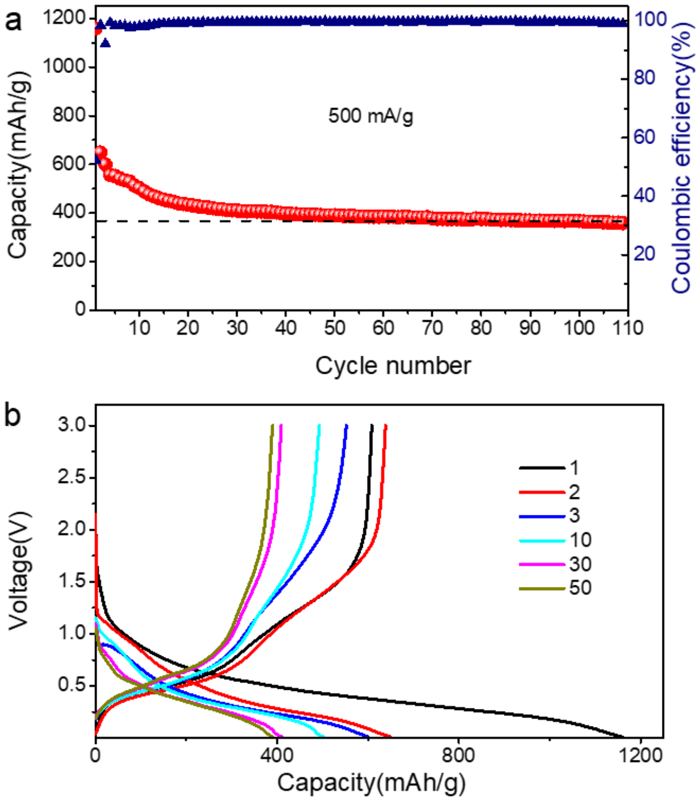

Fig. S18. Electrochemical performance of multi-layered STCT composites.

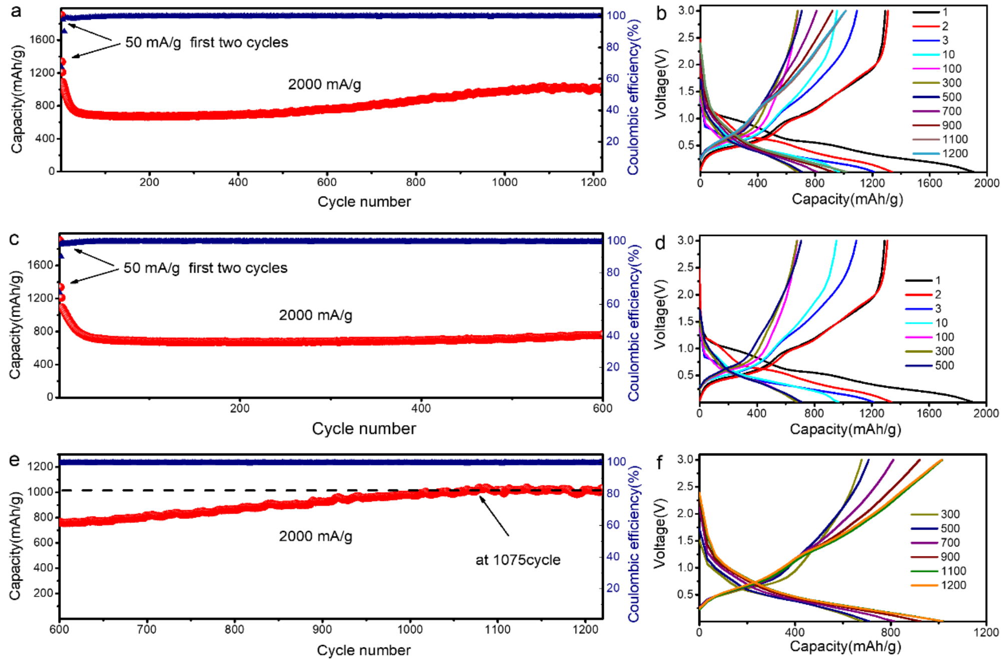

Fig. S19. Supplementary long cycling performance of STCT composites at 2000 mA/g. (a) The whole cycling performance. (b) The whole typical certain cycles galvanostatic discharge/charge curves. (c)-(f) Partial magnified cycling performance or galvanostatic discharge/charge curves.

The result show that the capacity has a tendency of increasing and can be stabilized at about1075th cycle.

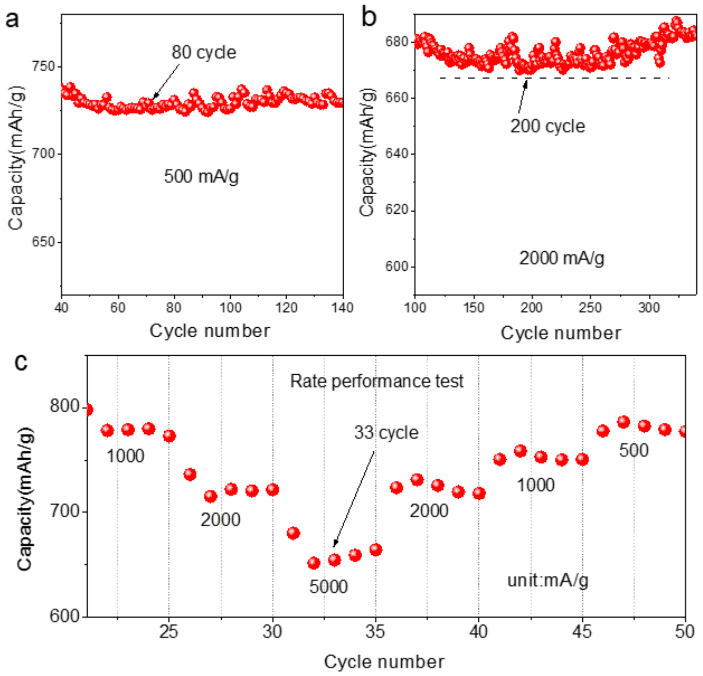

Fig. S20. Analysis of capacity rising phenomenon from the perspective of cycle number.

Table S1. Comprehensive analysis of capacity rising phenomenon from the perspective of cycle number and testing time.

Current density (mA)	Cycle number	Testing time (h)
500	80	356 h
2000	200	202 h
Rate performance test	33	452 h

Electrochemical measurements show the capacity increasing phenomenon happen at various current densities with the cycle going. The larger the current density, the more serious the volume expansion of active materials and more drastic ions insertion/desertion, as a result, the increasing phenomenon of capacity is more obvious and earlier in testing time.

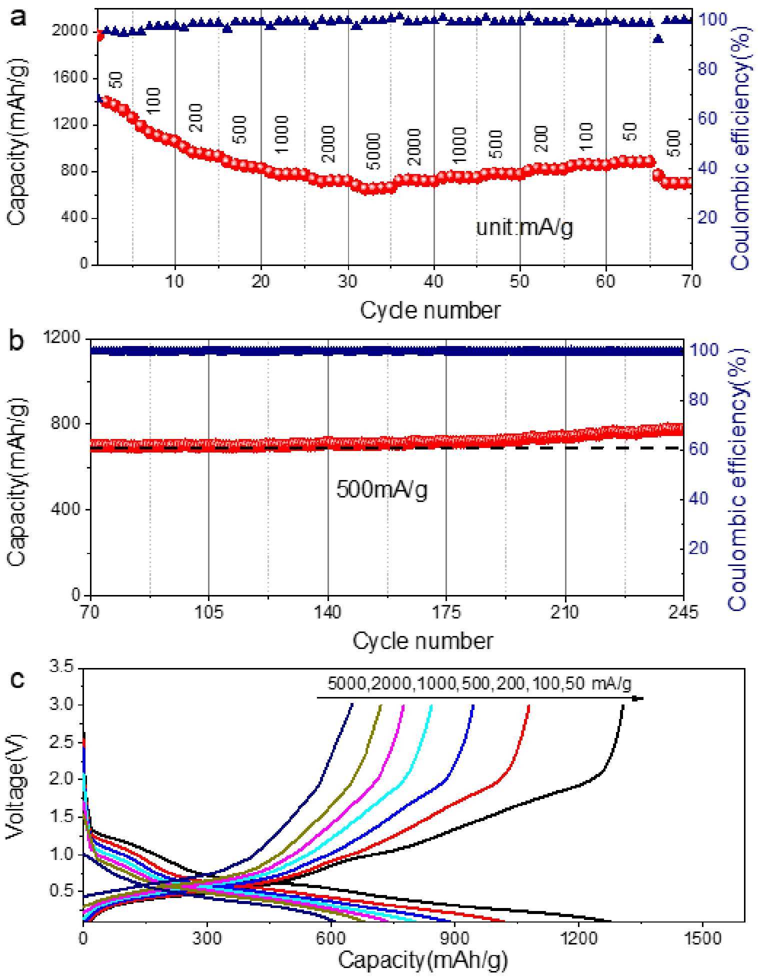

Fig. S21. Supplementary electrochemical performance of STCT composites. (a) Rate performance at different current densities from first time to 70th cycles. (b) Rate performance at subsequent cycles from 70th to 245th. (c) Typical galvanostatic discharge/charge curves at different current density from 50 mA/g to 5000 mA/g.

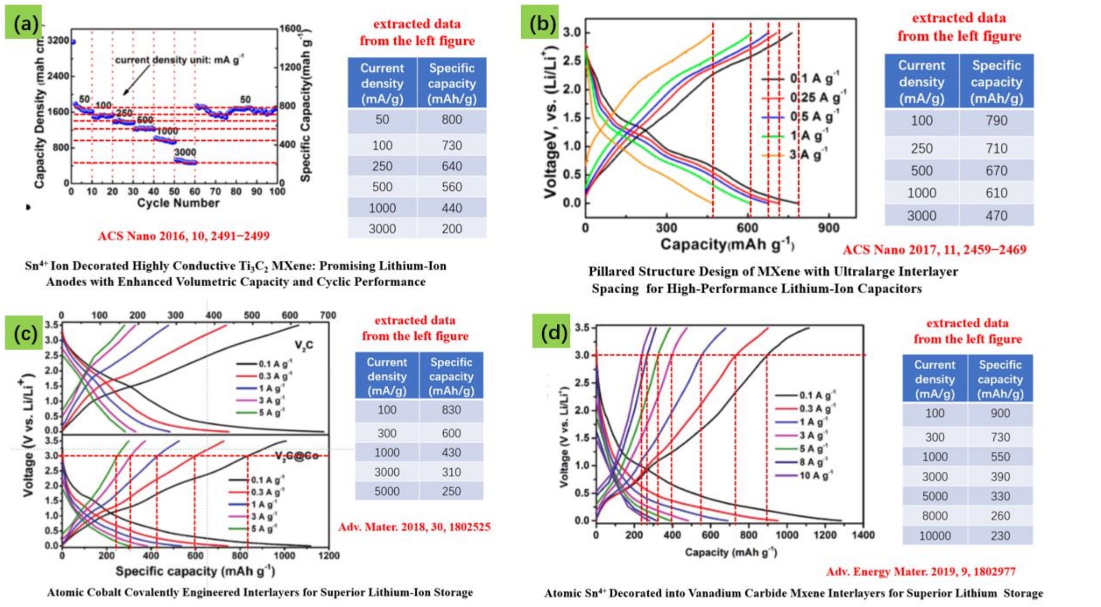

Fig. S22. Analysis of rate performance at reported literature about pillared-MXenes composites to get approximate specific capacity at various current densities.

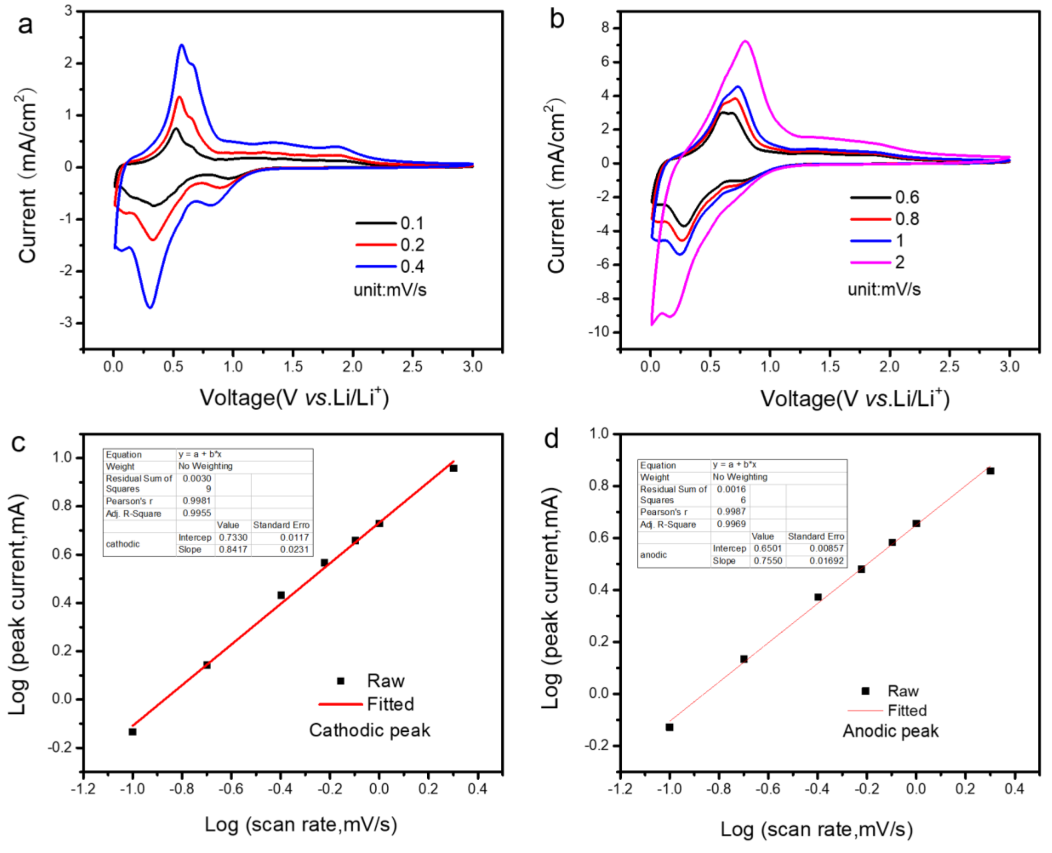

Fig. S23. (a)-(b) Detailed CV curves of the STCT composites electrode at various scan rates. (c)-(d) the relationship between the peak currents and scan rates.

Table S2. Comparison of the composite performances with other MXene-based materials reported.

Materials	Current density	Specific capacity (mAh/g)	Cycle numbers	Year/References
PVP-Sn(IV)@Ti_3C_2	500 mA/g	544	200	2016/[S1]
CTAB−Sn(IV)@Ti_3C_2	1000 mA/g	506	250	2017/[S2]
Sn-intercalated V_2C MXene	300 mA/g	600	1000	2019/[S3]
Co-intercalated V_2C MXene	300 mA/g	520	16000	2018/[S4]
Sn/SnO_x@MXene	50 mA/g	594.2	200	2019/[S5]
SnO_2-MXene	100 mA/g	360	200	2016[S6]
SnO_2@MXene	1000 mA/g	530	500	2018[S7]
HfO_2/SnO_2/MXene	500 mA/g	843	50	2018[S8]
SnO_2 QDs@$Ti_3C_2T_x$	500 mA/g	500	700	2018[S9]
p-$Ti_3C_2T_x$ /CNT	0.5 C	500	100	2016/[S10]
TNDs and P composites	100 mA/g	600	150	2018/[S11]
MXenes-TiO_2 –RGO	100 mA/g	167.5	100	2018/[S12]
MXene−rGO hybrid film	1000 mA/g	212	1000	2018/[S13]
STCT composites	2000	1000+	1000+ Without decay	This work

Supplementary References

[S1] J. Luo, X. Tao, J. Zhang, Y. Xia, W. Zhang. Sn($^{4+}$) Ion Decorated Highly Conductive Ti3c2 MXene: Promising Lithium-Ion Anodes with Enhanced Volumetric Capacity and Cyclic Performance. ACS Nano. **10**(2), 2491-2499 (2016). https://doi.org/10.1021/acsnano.5b07333

[S2] J. Luo, W. Zhang, H. Yuan, C. Jin, L. Zhang, H. Huang, C. Liang, Y. Xia, J. Zhang, Y. Gan. Pillared Structure Design of MXene with Ultralarge Interlayer Spacing for High-Performance Lithium-Ion Capacitors. ACS Nano. **11**(3), 2459-2469 (2017). https://doi.org/10.1021/acsnano.6b07668

[S3] C. Wang, S. Chen, H. Xie, S. Wei, C. Wu, L. Song. Atomic Sn^{4+} Decorated into Vanadium Carbide MXene Interlayers for Superior Lithium Storage. Advanced Energy Materials. **9**(4), 1802977 (2019). https://doi.org/10.1002/aenm.201802977

[S4] C. Wang, X. Hui, C. Shuangming, G. Binghui, L. Daobin, W. Chuanqiang, X. Wenjie, C. Wangsheng, B. Ganguli, A. P. M. Atomic Cobalt Covalently Engineered Interlayers for Superior Lithium-Ion Storage. Advanced Materials. **30**(32), 1802525 (2018). https://doi.org/10.1002/adma.201802525

[S5] D. Zuo, S. Song, C. An, L. Tang, Z. He, J. Zheng. Synthesis of Sandwich-Like

Structured Sn/SnO_x@MXene Composite through in-Situ Growth for Highly Reversible Lithium Storage. Nano Energy. **62**, 401-409 (2019). https://doi.org/10.1016/j.nanoen.2019.05.062

[S6] F. Wang, Z. Wang, J. Zhu, H. Yang, X. Chen, L. Wang, C. Yang. Facile Synthesis SnO_2 Nanoparticle-Modified Ti_3C_2 MXene Nanocomposites for Enhanced Lithium Storage Application. Journal of Materials Science. **52**(7), 1-10 (2016). https://doi.org/10.1007/s10853-016-0369-7

[S7] Y. T. Liu, P. Zhang, N. Sun, B. Anasori, Q. Z. Zhu, H. Liu, Y. Gogotsi, B. Xu. Self-Assembly of Transition Metal Oxide Nanostructures on MXene Nanosheets for Fast and Stable Lithium Storage. Advanced Materials. **30**(23), 1707334 (2018). https://doi.org/10.1002/adma.201707334

[S8] B. Ahmed, D. H. Anjum, Y. Gogotsi, H. N. Alshareef. Atomic Layer Deposition of SnO_2 on MXene for Li-Ion Battery Anodes. Nano Energy. **34**, 249-256 (2017). https://doi.org/10.1016/j.nanoen.2017.02.043

[S9] J. Xiong, L. Pan, H. Wang, F. Du, Y. Chen, J. Yang, C. J. Zhang. Synergistically Enhanced Lithium Storage Performance Based on Titanium Carbide Nanosheets (MXene) Backbone and SnO_2 Quantum Dots. Electrochim. Acta. **268**, 503-511 (2018). https://doi.org/10.1016/j.electacta.2018.02.090

[S10] C. E. Ren, M. Q. Zhao, T. Makaryan, J. Halim, M. Boota, S. Kota, B. Anasori, M. Barsoum, Y. Gogotsi. Porous Two-Dimensional Transition Metal Carbide (MXene) Flakes for High-Performance Li-Ion Storage. ChemElectroChem. **3**(5), 689-693 (2016). https://doi.org/10.1002/celc.201600059

[S11] T. Zhang, J. Xi, G. Li, Q. Yao, J. Y. Lee. A Red-Phosphorous-Assisted Ball-Milling Synthesis of Few-Layered $Ti_3C_2T_x$ (MXene) Nanodot Composite. ChemNanoMat. **4**, 56-60 (2018). https://doi.org/10.1002/cnma.201700232

[S12] R. Wang, S. Wang, Y. Zhang, D. Jin, X. Tao, L. Zhang. Graphene Coupled Ti_3C_2 MXenes-Derived TiO_2 Mesostructure: Promising Sodium-Ion Capacitor Anode with Fast Ion Storage and Long-Term Cycling. Journal of Materials Chemistry A. **6**(3), 1017-1027 (2018). https://doi.org/10.1039/C7TA09153B

[S13] Z. Ma, X. Zhou, W. Deng, D. Lei, Z. Liu. 3D Porous MXene (Ti_3C_2)/Reduced Graphene Oxide Hybrid Films for Advanced Lithium Storage. ACS Applied Materials & Interfaces. **10**(4), 3634-3643 (2018). https://doi.org/10.1021/acsami.7b17386
